# Odontoid fractures above C2 to pelvis posterior instrumented fusions: a single center’s 11-year experience

**DOI:** 10.1007/s43390-023-00800-z

**Published:** 2023-12-29

**Authors:** Perry Lim, Aaron J. Clark, Vedat Deviren, Sigurd H. Berven, Shane Burch, Christopher P. Ames, Alekos A. Theologis

**Affiliations:** 1 Department of Orthopaedic Surgery, University of California-San Francisco (UCSF), 500 Parnassus Ave, MUW 3 Floor, San Francisco, CA 94143 USA; 2 Department of Neurological Surgery, UCSF, San Francisco, CA USA

**Keywords:** Adult spinal deformity, C2-pelvis, Proximal junctional fracture, Dens fracture

## Abstract

**Purpose:**

To define the prevalence, characteristics, and treatment approach for proximal junction failure secondary to odontoid fractures in patients with prior C2-pelvis posterior instrumented fusions (PSF).

**Methods:**

A single institution’s database was queried for multi-level fusions (6+ levels), including a cervical component. Posterior instrumentation from C2-pelvis and minimum 6-month follow-up was inclusion criteria. Patients who sustained dens fractures were identified; each fracture was subdivided based on Anderson & D’Alonzo and Grauer’s classifications. Comparisons between the groups were performed using Chi-square and *T* tests.

**Results:**

80 patients (71.3% female; average age 68.1 ± 8.1 years; 45.0% osteoporosis) were included. Average follow-up was 59.8 ± 42.7 months. Six patients (7.5%) suffered an odontoid fracture post-operatively. Cause of fracture in all patients was a mechanical fall. Average time to fracture was 23 ± 23.1 months. Average follow-up after initiation of fracture management was 5.84 ± 4 years (minimum 1 year). Three patients sustained type IIA fractures one of which had a concomitant unilateral C2 pars fracture. Three patients sustained comminuted type III fractures with concomitant unilateral C2 pars fractures. Initial treatment included operative care in 2 patients, and an attempt at non-operative care in 4. Non-operative care failed in 75% of patients who ultimately required revision with proximal extension. All patients with a concomitant pars fracture had failure of non-operative care. Patients with an intact pars were more stable, but 50% required revision for pain.

**Conclusions:**

In this 11-year experience at a single institution, the prevalence of odontoid fractures above a C2-pelvis PSF was 7.5%. Fracture morphology varied, but 50% were complex, comminuted C2 body fractures with concomitant pars fractures. While nonoperative management may be suitable for type II fractures with simple patterns, more complex and unstable fractures likely benefit from upfront surgical intervention to prevent fracture displacement and neural compression. As all fractures occurred secondary to a mechanical fall, inpatient and community measures aimed to minimize risk and prevent mechanical falls would be beneficial in this high-risk group.

## Introduction

Adult spinal deformity (ASD) is a highly heterogeneous disorder that can result in considerable pain and functional disability [1–3]. Surgical management for ASD has demonstrated cost effectiveness and beneficial outcomes compared to nonoperative management [1, 3–7]. The mainstay of ASD surgical management consists of multi-level posterior instrumented fusions of the thoracolumbar spine or cervicothoracic spine. Less frequently, patients may require index or revision operations that span from C2 to the pelvis. This may be indicated for severe concomitant cervicothoracic and thoracolumbar deformities, for proximal junctional failure (PJF) following prior upper thoracic–pelvis posterior instrumented fusions, or for distal junctional failures following prior cervicothoracic posterior instrumented fusions. While prior reports have demonstrated that cervical to pelvis posterior instrumented fusions can provide considerable improvement in function [8], rigidity of the entire spine creates junctional stresses at C2 that increases the risk of dens fractures.

Proximal junctional failure following long thoracolumbar posterior instrumented fusions remains a vexing phenomenon that can jeopardize outcomes, result in considerable morbidity, and often necessitate revision operations [9–12]. While there is a plethora of knowledge regarding PJF following thoracolumbar fusions, there is a dearth of information regarding proximal junctional dens fractures [9–20]. As such, the aim of this study is to define the prevalence, characteristics, and treatment approach for PJF secondary to odontoid fractures in patients with prior C2-pelvis posterior instrumented fusions.

## Methods

**Fig. 1:**
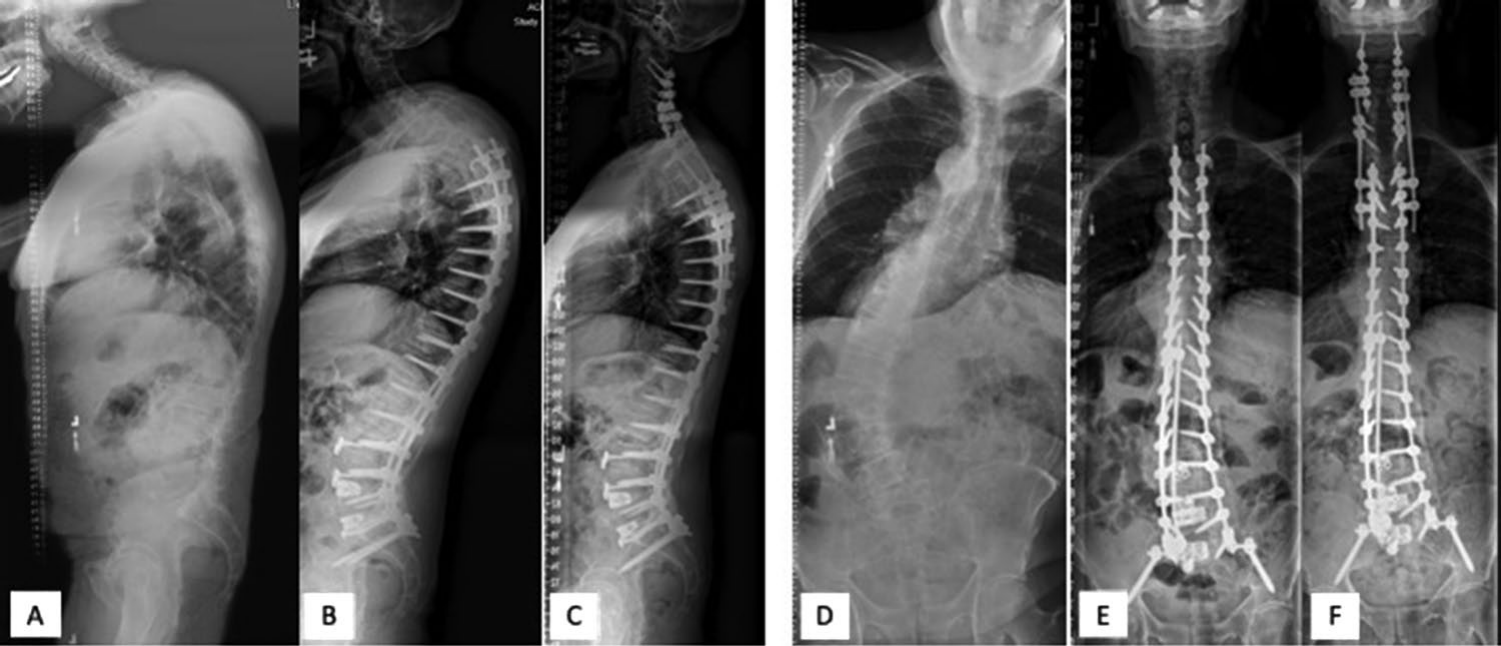
Representative radiographic example of a patient who underwent C2-pelvis posterior instrumented fusion for adult spinal deformity. A 67-year-old male with concomitant thoracolumbar scoliosis and cervicothoracic kyphosis (**A, D**) who was treated in a staged approach. First, he underwent an L3–S1 anterior lumbar interbody fusion and T3-pelvis posterior instrumented fusion (**B, E**) to address the thoracolumbar scoliosis. Three months later, an extension of posterior instrumentation to C2 with C7–T4 posterior column osteotomies was performed to address the persistent cervicothoracic deformity (**C, F**)

After Institutional Review Board approval, a surgical database between July 2012 and March 2023 was queried for patients who underwent multi-level posterior instrumented fusions consisting of ≥ 6 levels and including the cervical spine. Posterior segmental instrumentation from C2 to the pelvis (Fig. 1) and a minimum of 6 months of follow-up from the index operation were used as inclusion criteria. Patient demographics, including age, sex, body mass index (BMI), comorbidities, and the Charlson Comorbidity Index (CCI) [21], were recorded. Other clinical and surgical parameters included estimated blood loss (EBL), hospital length of stay (H-LOS), type of operation resulting in the C2-pelvis instrumentation (i.e., primary C2-pelvis PSF, extension of subaxial cervical–pelvis PSF to C2, extension of thoracic–pelvis PSF to C2, extension of C2-thoracic PSF to pelvis), use of a 3-column osteotomy (3CO), and post-operative complications.

Patients who sustained proximal junctional dens fractures as a complication post-operatively were identified by chart review. The diagnosis of each fracture was confirmed radiographically and verified by a fellowship-trained spinal surgeon from cervical computed tomography (CT) scans. Data regarding their clinical courses following fracture were assessed and the following were recorded: mechanism of injury, initial treatment modality (operative vs. nonoperative), date of revision operation relative to index operation, follow-up after definitive fracture management, reason for revision (i.e., pain, neurological symptoms, fracture displacement, etc.), and type of revision operation. In addition, cervical CT scans were reviewed and used to classify the type of dens fracture. Each fracture was classified based on the Anderson & D’Alonzo classification system (Type I—tip; Type II—base; Type III—body) [22]. Type II fractures’ morphology were further sub-classified based on the Grauer classification (Type A—horizontal fracture line; Type B—oblique fracture line from anterior/superior to posterior/inferior; Type C—oblique fracture line from anterior/inferior to posterior/superior—“reverse obliquity”) [23]. Fracture union was assessed with CT scans after 1 year following definitive management treatment.

Patient characteristics and revisions were compared between patients with and without a proximal junctional dens fracture. For categorical variables, χ^2^ tests were used to compare distributions. For continuous variables, student *T* tests were used to investigate differences in the distribution between the fracture and non-fracture cohorts. The level of significance was set at *p* < 0.05 for all tests. All data analysis performed utilized the Analyze-it software package in conjunction with Microsoft Excel.

## Results

### Patient cohort (Table 1)

**Table 1:** Comparison of characteristics between patients with and without proximal junctional odontoid fractures

Parameter	Entire cohort (n = 80)	+ Odontoid Frx (*n* = 6)	No Odontoid Frx (*n* = 74)	*p*
Age (years)	68.1 ± 8.1	71.8 ± 9.2	68.0 ± 8.2	0.23
Sex				
Male Female	23 57	0 6	23 51	0.11
BMI	28.7 ± 5.7	23.9 ± 4.1	29.1 ± 5.6	**0.03**
CCI	3.7 ± 1.7	3.2 ± 1.5	3.7 ± 1.7	0.43
Osteoporosis	36 (45%)	3 (50%)	33 (45%)	0.80
Estimated blood loss (mL)	1671 ± 1471 (300–8000)	1642 ± 1058 (400–3500)	1673 ± 1505 (300–8000)	0.96
Hospital length of stay (days)	12.9 ± 11.1	10.3 ± 7.1	13.1 ± 11.3	0.57
C2-pelvis operation type				
Primary C2-pelvis Extend Cervical–Pelvis to C2 Extend Thoracic–Pelvis to C2 Extend C2-Thoracic to pelvis	6 (7.5%) 10 (12.5%) 44 (55%) 20 (25%)	0 (0%) 1 (16.7%) 2 (33.3%) 3 (50%)	6 (8.1%) 9 (12.2%) 42 (56.7%) 17 (23.0%)	0.22
3CO	45 (56.3%)	2 (33.3%)	43 (58.1%)	0.24

Bold represents statistical significance (*p* < 0.05)

*Frx* fracture, *BMI* body mass index, *CCI* Charlson Comorbidity Index, *3CO* 3-column osteotomy

Eighty patients had C2-pelvis posterior instrumented fusions and were included for analysis. Average age was 68.1 ± 8.1 years. The majority (71.3%) were female. Average CCI was 3.7 ± 1.7 and average BMI was 28.7 ± 5.7. Of the 80 patients, 45.0% had osteoporosis. Average EBL for index C2-pelvis PSF was 1671 ± 1,471 mL (300–8000 mL). While occurrence of any complication was common (n = 51), complications were variable and each in isolation was uncommon. Neurological complications included cervical palsy (3.8%), brachial plexopathy (2.5%), epidural hematoma (2.5%), Bell’s palsy (1.4%), stroke (1.3%), and cauda equina syndrome (1.3%). Pulmonary complications included aspiration (2.5%), pneumothorax (1.3%), and pulmonary embolism (2.5%). Cardiac complications included atrial fibrillation (5.0%), hypotension (3.8%), myocardial infarction (2.7%), pulseless electrical activity (2.5%), and type II heart block (1.3%). Hematologic complications included deep venous thrombosis (2.5%). Infectious complications included vertebral osteomyelitis (1.3%), urinary tract infection (3.8%), Clostridium difficile (2.5%), and deep wound infection (1.3%). Alimentary tract complications included dysphagia (5.0%), perforated diverticulitis (1.4%), and shock liver (1.3%). Renal complications included acute kidney injury (1.3%) and hyponatremia (1.4%). Average follow-up for all patients was 59.8 ± 42.7 months.

### Dens fractures (Tables 1 and 2)

**Table 2:** Characteristics of patients who sustained proximal junctional odontoid fractures

	#1	#2	#3	#4	#5	#6
Age (years)	69	79	82	71	61	74
BMI	22.2	29	17	24	26	25
CCI	2	4	4	5	1	3
Osteoporosis	Yes	Yes	No	No	Yes	No
Initial C2 instrumentation	B/L pars screws	B/L pedicle screws	B/L pars screws	B/L pars screws	B/L pars screws	B/L translaminar screws
Time to fracture (months)	1.1	31.1	1.2	10.0	59.7	34.8
Mechanism of Frx	Fall	Fall	Fall	Fall	Fall	Fall
Frx classification						
Anderson/D’Alonzo Grauer	II A	III + unilateral C2 pars frx N/A	II A	III + unilateral C2 pars frx N/A	II + unilateral C2 pars frx A	III + unilateral C2 pars frx N/A
Failure of nonop	No	Yes	No	Yes	Yes	No
Treatment	Non-op	Extension to C1	Extension to C1	Extension to occiput	Extension to occiput	Extension to occiput
Reason for revision	N/A	Pain, Frx displacement	Pain	Pain, Frx displacement	Frx, displacement; torticollis; C1–2 stenosis w/myelopathy	Pain

*Frx* fracture, *BMI* body mass index, *CCI* Charlson Comorbidity Index, *B/L* bilateral

Six patients (7.5%) suffered a proximal junctional odontoid fracture post-operatively. Of the odontoid fracture cohort, the average age was 71.8 ± 9.2 years, CCI was 3.2 ± 1.5, and BMI was 23.9 ± 4.1. All six patients in the cohort were female with 50% having osteoporosis. Patients who sustained odontoid fractures had statistically lower BMIs compared to non-fracture patients (Table 1)**.** There were no significant differences between the two groups in regards to age, sex, CCI, osteoporosis status, EBL, H-LOS, type of index operation, and use of 3CO (*p* > 0.05) (Table 1).

**Fig. 2:**
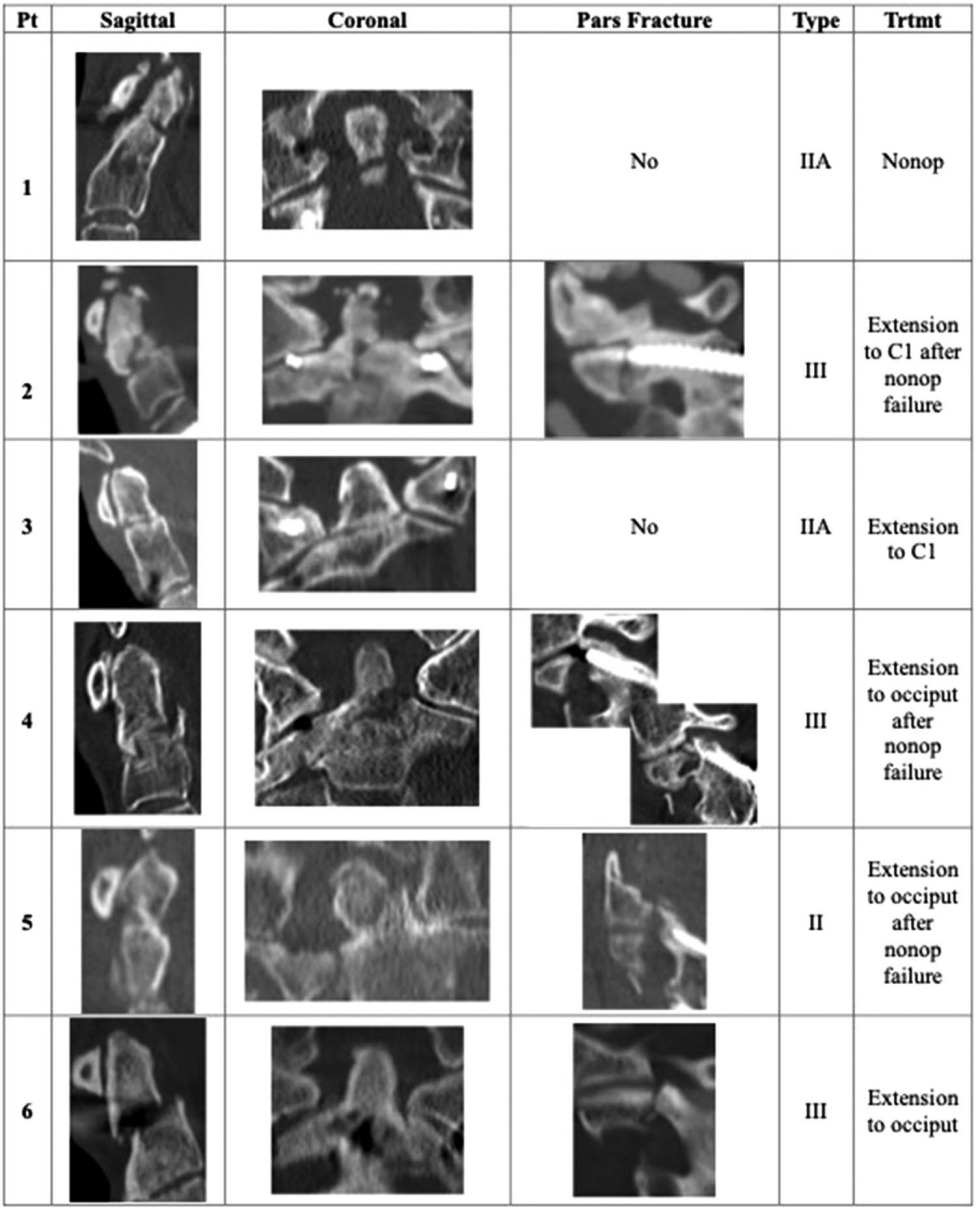
Fracture characteristics and morphology of proximal junctional dens fractures for each patient

The initial C2 instrumentation for the fracture cohort consisted of four (66.6%) bilateral pars screws, one (16.6%) bilateral pedicle screw, and one (16.6%) bilateral translaminar screw (Table 2). Cause of fracture in all patients was a mechanical fall. Average time to fracture was 23 ± 23.1 months. Fracture morphology varied, but 50% were complex, comminuted fractures of the C2 body (type III) with concomitant pars fractures (Fig. 2). Three patients sustained type IIA fractures, and one had a concomitant unilateral C2 pars fracture (Fig. 2). Three patients sustained comminuted type III fractures with concomitant unilateral C2 pars fractures (Fig. 2).

**Fig. 3:**
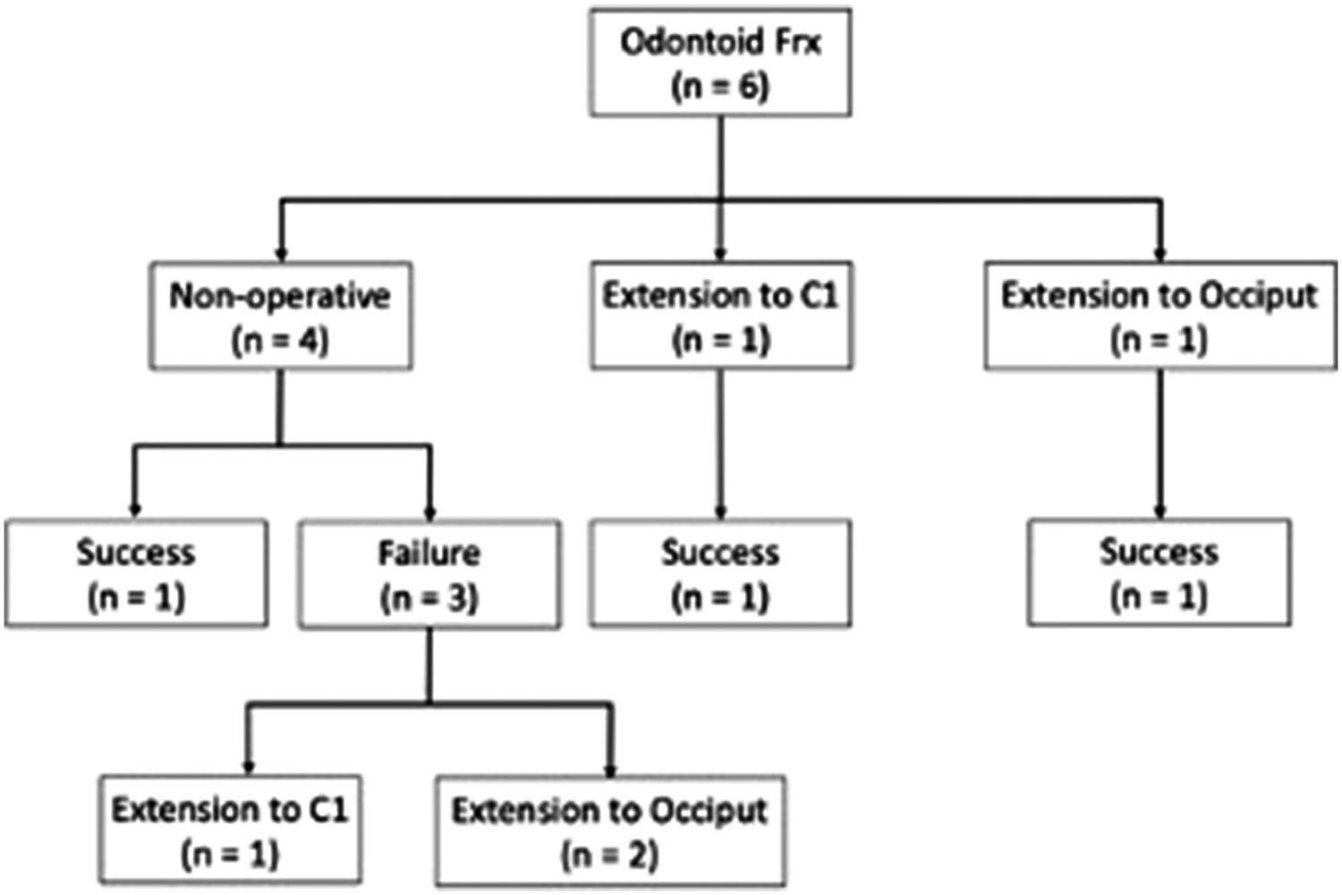
Schematic of management of proximal junctional odontoid fractures. “Success” of treatment is defined as fracture union as assessed by CT scan at least 1 year post-operatively

Three patients achieved fracture union with initial management, which included a collar, proximal extension to C1, or proximal extension to the occiput (Fig. 3). The other three patients were initially treated non-operatively, but later required proximal extension to the occiput or C1 for continued pain, fracture displacement, and/or C1–2 stenosis with associated cervical myelopathy (Fig. 3). The nonoperative failures were in patients with comminuted Type III or Type IIA fractures with unilateral C2 pars fractures. The 5 revision operations to address the odontoid fractures had an average EBL of 262.5 ± 188.7 mL (50–500 mL) and an average H-LOS of 5.8 ± 1.7 days. None of the revision surgeries experienced a post-operative complication. All fractures healed after surgery. Average follow-up after definitive fracture management was 5.84 ± 4 years.

## Discussion

Proximal junctional failures following posterior instrumented fusions for adult spinal deformity remain challenging clinical and surgical dilemmas. An odontoid fracture cranial to a prior C2-pelvis posterior instrumented fusions represents a unique subset of PJFs that is poorly understood. In this study, we have presented for the first time the prevalence, characteristics, and treatment approaches for PJF secondary to odontoid fractures in patients with prior C2-pelvis posterior instrumented fusions at a single institution over an 11-year timeframe. There were 3 major findings to this study: (1) the prevalence of proximal junctional dens fractures was 7.5%; (2) dens fracture morphology varied, but 50% were complex, comminuted C2 body fractures (type III) with concomitant pars fractures; and (3) nonoperative management was not suitable for comminuted dens fractures with concomitant C2 pars fractures. These results complement, expand upon, and bridge the disparate fields of spinal trauma and spinal deformity.

Optimal management of dens fractures is a topic of considerable interest and controversy. This is particularly so for type II dens fractures in elderly patients. While several reports support operative management given high risk of nonunion and jeopardized survival with nonoperative management [24, 25], others favor nonoperative management for elderly patients given increased mortality rates and complications with operative treatment [26]. For type III fractures, the literature appears in agreement that nonoperative management with external immobilization results in acceptable outcomes with union rates of 85–100% [27, 28]. Several of the considerations that go into treatment of isolated dens fracture in the elderly are applicable to our cohort, particularly risks of surgery in frail individuals and loss of rotation associated with immobilization of C1–2 and loss of flexion–extension with occipitocervical instrumentation. As C2-pelvis patients’ only spinal motion segments are C1–2 and occiput-C1, preservation of this motion through nonoperative care of a proximal junctional dens fracture would clearly be preferred if it were safe and effective. In our cohort, four out of six patients were treated nonoperatively initially. Of these four patients, three (75%) later required proximal extension to the occiput or C1 for continued pain, fracture displacement, rigid torticollis, and/or C1–2 stenosis with associated cervical myelopathy. It is important to note that all three nonoperative failures were in patients with comminuted Type III fractures with unilateral C2 pars fractures and the one successful outcome from nonoperative management was in a 69-year-old female who had a non-comminuted Type IIA fracture. Therefore, while nonoperative management may be suitable for type II fractures with simple fracture patterns, more complex and unstable fractures likely benefit from upfront surgical intervention to prevent fracture displacement, neural compression, and post-traumatic deformities, which may be more challenging and riskier to treat than the original injury. Despite the functional limitations from loss of range of motion secondary to immobilization of C1–2 and/or occiput-C1, the clinical, surgical, and economic consequences of a less-definitive treatment should be considered.

One upfront surgical intervention that can be considered to provide immediate stability, but preserve motion in the long term is to perform a proximal extension of instrumentation for fracture stabilization without fusion followed by removal of the instrumentation once the fracture heals. This approach has been previously demonstrated feasible, safe, and effective for isolated dens fractures as well as proximal junctional dens fractures [29–31]. Temporary fixation for isolated dens fractures was first reported by Han et al. who reported on 13 patients in whom dynamic CT scans demonstrated restoration of neck rotation compared to matched historic controls following dens fracture union and removal of temporary C1–2 fixation [31, 32]. In addition to restoration of motion, Guo et al. found that patients who had removal of C1–2 temporary instrumentation for dens fractures had significantly less neck pain, less neck stiffness, lower disability scores, greater satisfaction, and higher general functional scores compared to odontoid fractures treated with permanent posterior instrumented C1–2 fusions [30]. In 2017, Theologis et al*.* presented a case report of a 74-year-old female who was treated with extension of instrumentation to the occiput (no attempt at fusion was performed) for a displaced odontoid fracture with an unilateral C2 pars fracture after a mechanical fall 3 years after a C2-pelvis posterior instrumented fusion [29]. Post-operatively, severely limited neck range of motion was highly disabling to the patient, which was reflected in worsening of health-related quality of life scores [29]. The fracture healed uneventfully after which the instrumentation from the occiput and C1 were removed, which resulted in improvement of neck range of motion as well as minimal neck disability, no neck and arm pain, and outstanding general health functional scores [29].

In addition to management considerations, a discussion on prevention of these complicated junctional fractures is warranted. Intra-operative methods to consider to prevent proximal junctional dens fractures are placement of a prophylactic anterior odontoid screw with or without cement augmentation [33–35]. While an odontoid screw may theoretically prevent dens fractures, it may not be a viable strategy for a couple reasons. First, it would require a separate surgical anterior cervical approach, which would risk dysphagia in an elderly, frail cohort. Second, that a large proportion of our cohort’s fractures were comminuted body fractures with concomitant pars fractures raises the question of whether a simple odontoid screw would be efficacious in preventing these complicated fracture patterns. Another potential preventative measure could be cement augmentation of C2, which has been demonstrated safe and effective for treatment of cancerous lesions of the dens through minimally invasive anterior approaches (transoral and anterolateral) [36–43]. This theoretically could be performed pre-operatively, intra-operatively, or post-operatively in a staged fashion. These thoughts should neither be considered a recommendation nor advocacy for these techniques, but instead offer potential avenues to consider exploring in the future. Conversely, we favor promoting measures aimed to minimize risk of mechanical falls given that all out patients’ fractures occurred secondary to a mechanical fall [44]. A systematic review found that the following guidelines were strongly recommended to minimize falls: risk stratification, assessment tests for gait and balance, fracture and osteoporosis management, medication review, exercise promotion, environment modification, vision and footwear correction, referral to physiotherapy, and cardiovascular interventions [44].

The results of this study should be considered in the context of its limitations. The major limitations all stem primarily from the infrequent occurrence of patients being instrumented from C2 to the pelvis. Despite being an extremely high-volume adult spinal deformity center, only 80 patients with C2-pelvis posterior instrumentation constructs over an 11-year timeframe were performed (< 8 cases per year). As such, the resultant patient cohort of interest (i.e., proximal junctional dens fracture) was quite small and had considerable heterogeneity with regards to medical and surgical profiles and follow-up duration. In turn, our findings may not be generalizable and/or representative of the experience of the spine community as a whole. Furthermore, evolving practice patterns and inconsistent utilization of patient reported outcome measures over the 11-year study period rendered inclusion of validated clinical outcome measures not possible. In addition, while all patients received treatment for osteoporosis peri-operatively, the treatment approaches (i.e., duration of treatment, type of pharmacology, assessment of treatment “efficacy”) were quite variable given changing practice patterns. In addition to osteoporosis management, it is possible that other factors may have been involved in the development of the fractures, including, but not limited to, post-operative radiographic alignment parameters. However, given that all the fractures in our cohort occurred following mechanical falls, radiographic alignment parameters likely play a negligible role in the development of these types of adjacent fractures, and thus, there measurement was felt to be outside the scope of this study. While our minimum follow-up of 6 months may be considered incomplete, it encompasses all patients who may have developed acute PJF, as defined by the development of PJF within 6 months after operation [13, 45]. Furthermore, given that the vast majority of patients who present to other hospitals are commonly transferred back to our hospital for management given complexity of their previous spine surgeries, patients included in this study are likely a good representation of the prevalence of dens fractures in this unique patient cohort. Despite these limitations, this is the most robust cohort to be presented on patients who had prior C2-pelvis posterior instrumented fusions and the first study on proximal junctional dens fractures. As such, we anticipate that our observational findings will bring awareness to this challenging problem and will ideally be a launching pad from which multi-center retrospective and prospective investigations will be initiated to assist in fully appreciating the incidence, burden of disease, and optimal methods to prevent and treat this unique subset of PJF.

## Conclusions

In this 11-year experience at a single institution, the prevalence of odontoid fractures above a C2-pelvis PSF was 7.5%. Fracture morphology varied, but 50% were complex, comminuted C2 body fractures (type III) with concomitant pars fractures. While nonoperative management may be suitable for type II fractures with simple patterns, more complex and unstable fractures likely benefit from up front surgical intervention to prevent fracture displacement and neural compression. These results highlight the need for surgeons to be cognizant of the risk of odontoid fractures after C2-pelvis posterior instrumented fusions and provide some guidance on how to approach initial management. Importantly, as all fractures occurred secondary to a mechanical fall, inpatient and community measures aimed to minimize risk and prevent mechanical falls would be beneficial in this high-risk group. Additional clinical investigations, including multi-center data, will be helpful to fully appreciate the incidence, burden of disease, and optimal methods to prevent and treat this unique subset of PJF.

## Data Availability

The data that support the findings of this study are available from the corresponding author, AAT, upon reasonable request.
